# Impact of COVID-19 lockdown on physical activity, insomnia, and loneliness among Spanish women and men

**DOI:** 10.1038/s41598-023-30173-2

**Published:** 2023-02-20

**Authors:** Myriam Guerra-Balic, Carina S. González-González, Oriol Sansano-Nadal, Adriana López-Dóriga, Ming-Kai Chin, Kele Ding, Jingzhen Yang, J. Larry Durstine

**Affiliations:** 1 Department of Physical Activity and Sports Sciences, Faculty of Psychology, Education and Sports Sciences Blanquerna, University Ramon Llull, Barcelona, Spain; 2 Department of Computer Engineering and Systems, Women Studies Institute (IUEM), Universidad de La Laguna, La Laguna, Spain; 3 Oncology Data Analytics Program, Catalan Institute of Oncology, l’Hospitalet de Llobregat, Barcelona, Spain; 4 Foundation for Global Community Health, Las Vegas, USA; 5 School of Health Science, College of Education Health & Human Service, Kent State University, Kent, USA; 6 Center for Injury Research and Policy, The Abigail Wexner Research Institute, Nationwide Children’s Hospital, Columbus, USA; 7 Department of Exercise Science, Norman J. Arnold School of Public Health, University of South Carolina, Columbia, USA; 8 School of Health and Sport Sciences (EUSES), Rovira i Virgili University, Tarragona, Spain

**Keywords:** Health care, Risk factors

## Abstract

During COVID-19 pandemic, quality of living was impacted by social isolation, loneliness, and altered sleep habits. The aims of this study were (1) to examine the relationship between physical activity (PA) levels with insomnia and loneliness among adults during Spain’s first COVID-19 wave of lockdown and its impact on women and (2) to examine the digital technologic resources used to support both PA and other recreational activities in women. A cross-sectional design was used. An anonymous 15-min online survey was conducted in Spain to adults (≥ 18 years old) during the first COVID-19 lockdown, a 40-day period. A snowball distribution method was employed using personal email and social networks (Facebook, Whatsapp, Linkedin, Twitter). Variables studied included: socio-demographic items, insomnia, loneliness, PA, and digital technologic resources. A total of 996 adults (females = 663, 66.6%) completed the survey. Higher education levels were associated with greater PA levels (p-value < 0.001). Women presented with higher insomnia risk than men with low PA levels (OR = 1.9, CI = 1.25; 2.95). Living with family members or other individuals was related to lower insomnia risk. A strong correlation between medium–high PA levels was found with greater digital technology resources (DTS) than individuals with low PA levels. Females used significantly more DTS than males (p-value < 0.001). No significant associations between DTS were found with age or academic education level. PA levels, sex, and loneliness were related to insomnia risk. A strong correlation between PA and DTS use was observed. Participants with medium–high PA levels and females use them more than those with low PA levels and males. We recommend promoting the PA through digital technologies for women. This recommendation would also improve sleep disorders in women who present higher insomnia risks than men.

## Introduction

Regular PA is an effective strategy to reduce the psychological effects of the COVID-19 pandemic, which can help people cope with difficult times and fend off infections^1^. During the SARS-CoV-2 (COVID-19) pandemic, physical activity (PA) behaviors were altered around the world due to public health measures such as “lockdowns.” As a result, people are forced to stay at home, with a significant reduction in PA levels and increased sedentary behavior. Such a sudden change in PA behaviors and social isolation has had a significant negative impact not only on the physical health but also on the mental health of individuals^1,2^. Besides, taking into account that there is a relationship between the usage of digital equipment and the academic education level, it is interesting to note that, according to the National Institute of Statistics of Spain, for the adult population (aged 25–64 years old) the percentages for high academic level are 37,3% for men and 44,0% in women.

Existing evidence suggests that social isolation, defined as the absence of regular contact with family and friends and a lack of participation in social organizations, is associated with lower daily PA and increased sedentary lifestyles^3^. Social isolation may also lead to loneliness, a subjective experience of being isolated that has negative implications on people’s well-being and health^4,5^. While social isolation and loneliness are distinctly different variables, both increased during the COVID-19 lockdown and had a negative influence on health^6^. Banerjee and Rai found that the social restrictions during the COVID-19 pandemic limited social interactions, which could contribute to the risk of domestic inter-personal violence and boredom^7^. Pai and Vella (2021) in their rapid systematic review concluded that loneliness had an impact on the mental health and wellbeing of the general adult population during COVID-19^8^.

Another negative effect of the lockdown is altered sleep patterns, which leads to increased risk of sleep disorder. One of sleeping disorders associated with COVID-19 lockdown is insomnia, defined as difficulty with sleep initiation, duration, and consolidation of sleep quality^9^. Untreated insomnia can disrupt physical, mental, social, and emotional functioning, resulting in a range of physical and mental health problems including higher mortality and morbidity rates; increased depression and social isolation symptoms; increased negative cognitive, emotional, and social sequels; and poor performance in academic or professional achievements^10^. Marelli et al. (2021) studied sleep quality in university students and staff found that COVID-19 resulted in a worsening of sleep quality and insomnia symptoms, calling for education on circadian rhythms to avoid sleep disorders during periods of isolation^11^.

Though the pandemic has impacted health negatively, the pandemic has produced many positive health actions such as creating various online exercise programs^12^. Likewise, isolation is often offset by internet influences and can increase exercise adherence through internet social support^13^. Online programming during the pandemic has added value to leisure and physical and emotional well-being. Recent research from Europe, the United States, and Canada report that over 70% of people go online to get information, communicate with others, perform different tasks, and enjoy digital leisure^14^. Likewise, several Youtube channels offer free PA videos. For example, In Spain, the home exercise program 'En acción en casa' of Telemadrid offers daily PA sessions. Furthermore, specific mobile applications such as Vivifrail assess an individual’s physical fitness level and then provides a daily exercise program based on each clients’ initial fitness level. These classes are often pre-recorded and used asynchronously or are streamed using video streaming programs such as Zoom or Google Meet. Caputo & Reichert (2020) in their review study concluded that a decrease in PA levels due to social distancing measures is likely to contribute to an increased mental health burden related to the COVID-19 lockdown, suggesting that engaging in daily PA becomes more important than ever during the COVID-19 pandemic as PA is known to help maintain good mental health^15^. However, despite several recent systematic reviews were conducted to examine PA and mental health during their respective lockdowns across several populations, little data are available on the relationships between physical activity and insomnia, loneliness among Spanish people, even fewer studies reported such relationships among Spanish women. Given that COVID-19 may cast different shadows over the lives of women from men due to factors that intersect with gender roles and influence women and men’s vulnerability and resilience, it is important to understand the unequal experiences of COVID-19 lockdown that women have had^16,17^.

Concerning women, other authors have studied the relationship between PA performed outdoors and sleep quality in adult women, finding that there was a significant interaction (p = 0.04). They stated that both the time spent outdoors and the time of day can moderate this relationship. Moreover, they consider that light exposure, due to circadian rhythms can influence^20^. Galasso et al. analyzed the differences between women and men in attitudes and behaviors developed during the pandemic, and they found that women were more conscious about the severity and health risks of COVID-19 lockdown, so they better followed rules and recommendations for health prevention^16^. When focusing on sleep disorders, Guadagni et al. reported in females greater symptoms of insomnia than in males^30^.

In this study, we aimed to examine the relationship between PA levels with insomnia and loneliness among adults during Spain’s first COVID-19 wave of lockdown and its impact in women, and to examine the digital technologic resources used to support PA in women.

## Method

### Study design

This cross-sectional designed study utilized an open survey developed for use in eleven countries^18^. Data obtained for this study were collected from Spanish participants.

In Spain, lockdown started on March 14th, 2020, when the Spanish Government decreed a state of alert, forcing the entire Spanish population into total confinement in response to the first COVID-19 wave. Population confinement was extended twice in periods of 15 calendar days each with extensions in effect until June 21st, 2020. During this period and until the beginning of the de-escalation phases, people were not authorized to go out in the street except for basic necessities such as the acquisition of food, pharmaceutical products; assistance to health centers; or commuting to the workplace. De-escalation was done in phases beginning on May 4th, 2020, and restriction modification back towards the pre-escalation period was dependent on regional infection rates. Phase 0 began on May 4th, 2020, when individuals were allowed to participate in open air non-contact physical activity (PA). Phase 1 began on May 11th, 2020, when sports centers and fitness centers were opened to a 30% capacity and by appointment for activities not involving physical contact. The use of locker rooms was prohibited. Phase 2 began on May 25th, and in this phase, attending outdoor sporting events and activities with limited seating capacity was allowed. Also, during this time indoor sporting facilities were closed to the public. Phase 3 started on June 8th, 2020 and public attendance to sports facilities was limited to 1 person per 20 m^2^, and in gyms or fitness centers, use was limited to one-third capacity with no use of locker rooms.

### Study participants

Study participants represent a convenience sample and use the followed inclusion criteria: (1) adults older than 18 years old, (2) competent in the Spanish language, (3) living in Spain during the first COVID-19 wave of lockdown, (4) volunteer participation in the online survey, and (5) having internet connection available.

Participants were recruited by a snowball sampling utilizing personal email and social networks contacts (Facebook, WhatsApp, LinkedIn, Twitter). Subjects were sent a link for an anonymous and volunteer online survey as no incentives were offered. Data collection lasted for 40 days (from June 21st to July 30th, 2020). Country-level COVID-19 factors are presented in Ding et al.^18^. The data collection period coincided with phase 3 of Spain’s de-escalation beginning on May 4th, 2020, when restriction modifications were specific and depended on the rate of regional infection^19^. Lockdown restrictions were modified to allow for sporting events and PA which consisted of one person per 20 m^2^ when attending sporting events. These modifications were put in place to reduce personal contact and to reduce infection risk.

Information was obtained for the period during Spanish phases 1 and 2 when no possibilities existed for individuals to go outdoors (phase 1), and individuals were only allowed to go outdoors for specific hours (phase 2). May 1st, 2020 was the first time when people were allowed to go outdoors and walk or exercise alone or with persons with whom they lived. Gyms and fitness centers opened during phase 1 for individual training and only by appointment.

A total of 1075 surveys were obtained, and of these, 966 surveys were included in the final analysis excluding the 79 surveys (7.35%) with 90% or more of missing data or with non-Spanish respondents (some surveys were answered from other Spanish-speaking countries).

### Online questionnaire of global project

The survey was developed previously by a global project group^18^ and questionnaires were validated in Spanish through translation and back-translation. Specifically, survey questions were translated into Spanish and reviewed by an external group for consensus agreement. Surveys were again back-translated by an external group of bilingual English–Spanish speaking natives for accuracy. Questions were adapted for social characteristics such as academic education level and occupation salaries. The online survey was hosted on the survey platform of the University of Barcelona, which was not able to pre-select the sample. The final survey instrument included 73 questions, which took approximately 15 min to complete. Seventeen questions/items were analyzed for this study. These questions were mainly related to PA during the COVID-19 pandemic while two questions were related to loneliness and sleeping disorders.

### Study variables

Study variables were organized into five topical groups: socio-demographic items, insomnia, loneliness, PA, and digital technologic resources.

#### Socio-demographic variables

Sex, age, highest academic education level, living at home alone, having pets, and COVID-19 diagnoses. Due to inclusive language recommendations, when we referred to the study variables, we used the words female/male to describe the variable sex, considering the biological sex assigned at birth. We did not consider the diversity in sex, gender identity, and gender expression in this study. As, even in the survey the question related to sex included not only males and females but others or preferred not to answer, finally we only obtained enough answers from males and females, so we considered these words as synonymous with women/men.

#### PA

PA was measured using the International Physical Activity Questionnaire (IPAQ) short version^22,23^. The IPAQ short version has 9 items obtaining PA information from the last 7 days, was developed for monitoring PA and inactivity in adults aged 18–65 years^24^, and is an open-access questionnaire (https://www.ipaq.ki.se). The Compendium of Physical Activities developed by Ainsworth et al.^25^ was used to classify different types of physical activities: conditioning exercises (multiple exercises to include resistance exercises, flexibility, and balance), bicycling, dancing, running, walking, and different sporting activities^25^. PA performance included PA levels (vigorous, moderate, low), indoor or outdoor, type of PA, and PA as a coping strategy. A new variable was created based on variables that best described PA levels and depended on the number of days doing vigorous, moderate, or walking (considered low level) PA. Information was recodified into one variable defining the PA level as “High,” “Medium” or “Low” activity; people responding with None or 1 day walking and no vigorous PA were classified as Low activity, people who completed 2 or more days walking and less than 3 days of vigorous activity and were classified as Medium activity. Finally, people who did more than 3 days of vigorous activity were classified as High activity.

### Insomnia

Insomnia was measured using the Brief Resilient Coping Scale^20^. A 4-item scale with a 5-category symmetrical Likert scale was used^18^. Participants were asked to answer questions pertaining to their feelings during the COVID lockdown. Even though we used the Brief Resilient Coping Scale, which is a 4-item scale with a 5-category symmetrical Likert scale, for our study, we only analyzed the question “COVID kept me awake at night” (insomia) (never, rarely, sometimes, often, always; scoring 1,2,3,4, and 5, respectively).

#### Loneliness

Loneliness was obtained from the 12-item Herth Hope Index scale^21^. Each item uses a 4-point Likert-scale^16^. For this study, we only used the response choices for “I feel alone” (strongly disagree, somewhat disagree, somewhat agree, strongly agree; scoring 1, 2, 3 and 4, respectively).

#### Digital technologic resources

Digital technologic resources was measured using the questions asking about whether participants followed PA online programs and whether participants used online learning programs for entertainment.

### Statistical analysis

Chi-Square tests were performed to determine associations between categorical variables. Only the models based on Akaike’s criterion with significant associated factors were considered.

Descriptive analyses for all survey variables were completed. Spearman correlations were calculated for the number of days doing PA at different PA levels. Chi-Square tests were performed to determine bivariate associations between categorical variables. Multiple logistic regression models were computed to find significant factors associated with insomnia, loneliness, and PA. Specifically, models were built considering insomnia, loneliness, and PA as dependent variables, independent variables were other factors such as age ranges, sex, PA level comparison, and academic education level. Only the models based on Akaike’s criterion with significant associated factors were considered.

SPSS Statistics v26 (IBM Corp. USA) statistical package and R v3.6.0 software was used to analyze data.

### Ethical issues

This study followed the guidelines of the Declaration of Helsinki and was approved by the Institutional Review Board (IRB) at the University of Barcelona, Spain (reference: IRB00003099). The procedures met the requirements of the Spanish Organic Law 3/2018, December 5th, to protect personal data and digital rights. A code was given to each responder. Informed consent was obtained from participants.

On the first page of the online survey, participants found a question related to their age to ensure participants were eligible adults. Once age data was obtained, the subjects were given information concerning study participation responsibilities. Finally, participants accepted involvement with voluntary consent. If voluntary consent was not given, the remainder of the survey was blocked, and the participant was unable to continue. If participants accepted, the participant was given approximately 20 min to complete the survey. Participants were also informed that as a volunteer, they had the right to withdraw from the study at any time.

## Results

### Descriptive analysis

The sample consisted of 996 participants, which 663 (66.6%) were women and 288 (28.9%) were men. There were 45 participants from whom we still need an answer specifically to the questions analyzed in this study. Detailed descriptive analysis is included in the Supplemental Material, where variables are ordered by type of information depending on socio-demographic items, lockdown information, and PA items.

When comparing sex, a significant difference (p-value < 0.001) was found as men perform vigorous, moderate, and light PA more days than women. In addition, participants with a higher academic education level were significantly more physically active (p-value < 0.001).

Regarding altering PA from before to when lockdown ended, 54% of the study group declared they decreased PA levels while 22% of the study group increased PA moderately. Women corresponded to 68% of the ones that increased PA. Interestingly, PA levels of participants who were diagnosed and/or presented symptoms of the COVID-19 followed the same distribution as those individuals who did not suffer COVID-19, did not know, preferred not to answer, or N/A.

Concerning the type of PA completed during the lockdown, the most frequent response was conditioning exercise (55%), followed by walking (31%), dancing (14%), running (8%), bicycling (5%) and participating in general sports (3%). Most participants answered that they did not use online resources for entertainment (52%) but responded yes for other purposes (26%).

When asking about with whom participants performed PA, 51% answered they did it by themselves (63% of these were women), and 34% answered they exercised with family or other members (74% of these were women). The percentage of participants living alone was 8% while 24% felt alone. These data suggest that some individuals felt alone during the lockdown period even while living with family or others.

The number of participants performing PA indoor was 616 while 236 performed PA outdoors and 44 participants performed no PA. Depending on the region, city, or neighborhood that participants lived, indoor/outdoor restrictions may have been different and were under control of local organizations^26,27^.

### Bivariate associations

The number of days doing moderate and vigorous PA were highly correlated (Spearman Rho = 0.36; p-value < 0.00001). The recorded variable classifying PA in three levels (High, Medium, Low) was used for the following analysis. No significant bivariate associations between PA and insomnia or loneliness were found. However, a clear association existed between PA and digital technologic support (DTS). Participants with medium (74%) and high (67%) PA levels used more DTS than people with low PA levels (43%) (p-value < 0.001).

When the use of DTS and different variables were considered, females were found to use significantly more DTS (70%) than males (45%) (p-value < 0.001). No significant associations between DTS and age or academic education level were found.

**Figure 1:**
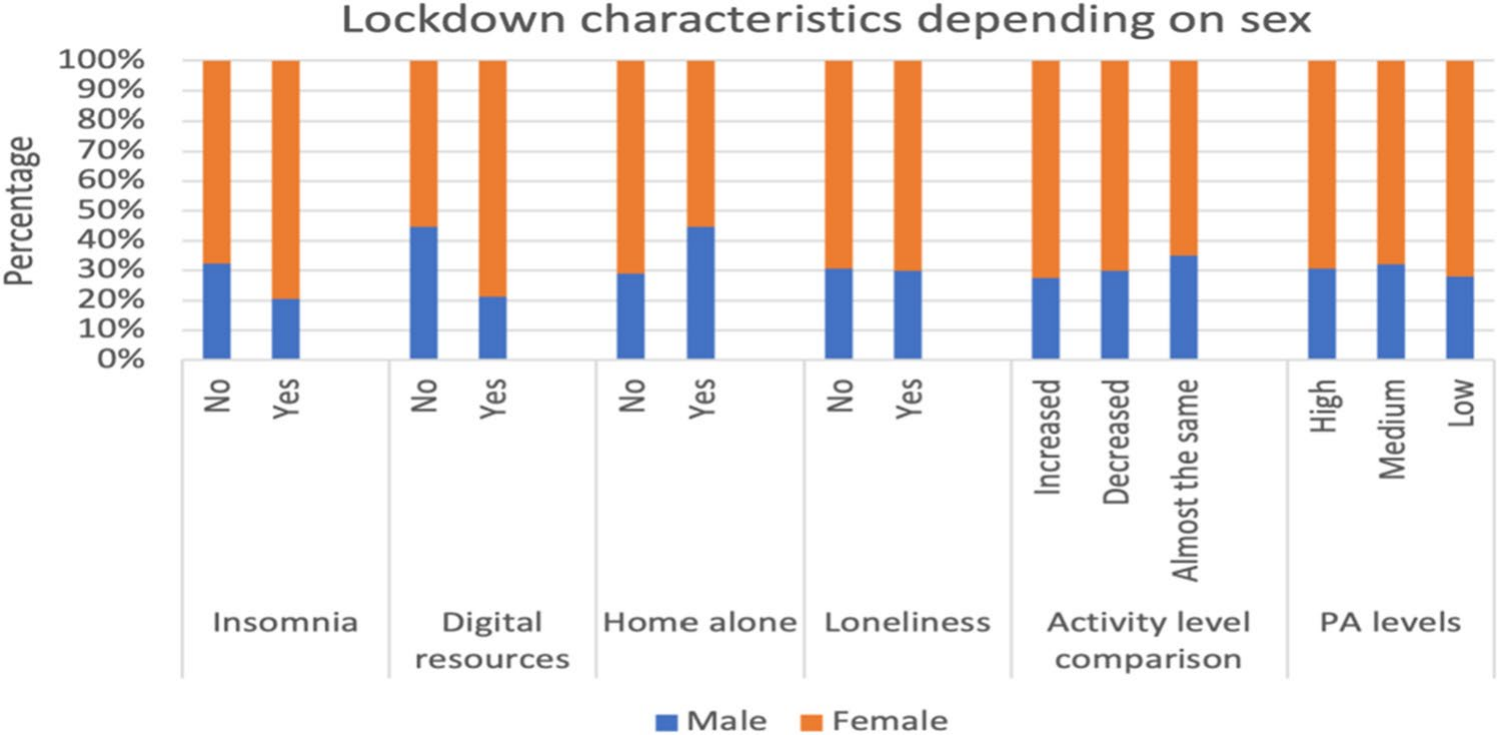
Barplot of cumulative percentages for males and females in studied factors. For each different answer in each studied factor, color bars show the proportion of males and females.

In Fig. 1, differences in sex percentages are shown regarding insomnia, use of digital resources, living home alone, loneliness and PA levels.

### Multiple logistic regression models

Multiple logistic regression models were built considering PA, insomnia and loneliness as dependent variables. Independent variables were other factors such as age ranges, sex, PA level comparison, and academic education level. Models showed no clear association factors with PA or loneliness. However, the model built with insomnia as an independent variable showed significant associations. Females were at higher insomnia risk than males with an OR = 1.9 (95%CI = 1.25; 2.95). When analyzing the question “PA level comparison”, females exhibiting decreased PA level during the lockdown according to their previous level were at higher insomnia risk compared to individuals that increased their physical activity level with an OR = 1.76 (95%CI = 1.11; 2.89, p-value = 0.018). Furthermore, living with family or other individuals was associated with a lower insomnia risk, OR = 0.519 (95%CI = 0.29; 0.95) (Table 1).

**Table 1:** Multiple logistic regression model for insomnia.

Characteristic	N	OR	95% CI	p-value
Sex				
Male	288	1		
Female	663	1.9	1.25; 2.96	0.0032
Home alone				
Lived by myself alone	78	1		
Live with family or other member(s)	889	0.52	0.29; 0.95	0.0274
Physical activity level comparison				
Increased	216	1		
Decreased	537	1.76	1.12; 2.89	0.018
Almost the same	175	0.97	0.51; 1.84	0.94
Don’t know …/hard to tell	21	1.28	0.28; 4.27	0.71

*OR* odds ratio, *CI* confidence interval.

## Discussion

The present study is based in a survey applied to study how the first COVID-19 pandemic lockdown impacted in an adult Spanish population, mainly focusing the COVID-19 impact on PA, insomnia and loneliness, especially on women. It also determined the digital technology resources used to support PA and other recreational activities.

The first study objective was to analyze adult PA levels related to insomnia and loneliness during the first Spanish COVID-19 quarantine wave. As results showed us, insomnia was more prevalent in females than males with an OR = 1.9 (95%CI = 1.25; 2.95, p-value = 0.0032). This is in contrast to Kokou Kpolou et al., who did not find gender associated with insomnia severity, however they did find a relationship with education level, as they found that participants with postgraduate academic levels presented lower levels of insomnia severity than participants with primary and college education levels^28^. This disagreement could be because of several considerations: the study was developed only during 13 days, it was developed in other country which might have different pandemic policies, and the number of participants was lower than in our study. In contrary, Falkingham et al. did find that women with young children experienced difficulty in sleeping during the pandemic lockdown^29^. The same happened in the study by Guadagny et al. (2020), which showed that sex and gender differences can condition the psychological and behavioral reactions to the COVID-19 pandemic, especially in women^30^.

Kokou Kpolou also showed that the worries related to the pandemic and the loneliness contribute to clinical insomnia^28^. Moreover, McLay et al. (2021) in a sample of elderly (> 65 years of age) showed how loneliness, social isolation and health concerns are related and may produce sleep disorders, especially in women, and they found that those who live alone and were socially isolated experienced insufficient sleep^31^. Both Kokou Kpolou and McLay et al.^28,31^ agreed with our findings on loneliness and insomnia.

Chang et al. (2013) observed that there was a relationship between regular PA and decreased insomnia symptoms^32^. Moreover, it has been shown that very low and very high levels of PA have been associated with sleep disorders^33^. In our study, with low PA, a higher insomnia risk was found with an OR = 1.76 (95%CI = 1.11; 2.89, p-value = 0.018). A similar study by Murray et al.^20^ specifically developed with a sample of 360 adult women, analyzed the role of PA and time outdoors in predicting sleep health, and found a positive significant interaction (p-value < 0.05) between moderate-to-vigorous PA and total sleep time. Nevertheless, they highlighted that there could be a difference if women exercise outdoors. Unfortunately, we did not analyze this condition. Another study developed by Sonnega et al. (2021) also suggested that PA can have moderate benefits for sleep in older adults^34^.

The loneliness and insomnia analyses results show that when living with family members or other individuals, an association was found for lower insomnia risk, OR = 0.519 (95%CI = 0.29; 0.95), which matches with what McLay showed^31^. Nevertheless, models from our study indicate no clear association with PA or loneliness with insomnia. In relation to the differences between sex, another study^35^ reported differences, where men were more physically active than women (65,5% vs. 53,9% respectively). This information matches with our findings.

It has been shown that digital platforms can help to meet MVPA during the COVID-19 lockdown restrictions when exercising outside the home is forbidden^36^. So, a second objective of our study was to consider if PA performed was supported by digital technologic resources. What we found was that women used significantly more DTS than men (p-value < 0.001) with 70% of women using DTS versus 45% for men. This matches with what Parker et al. described, as in their sample majority users were female. Nevertheless, Parker et al. found that two-thirds of the sample had a tertiary academic degree, which is contrary to what we found, as no association between age or academic education level with DTS was found in our study. This could be because Parker et al. included in their study not only adults but also adolescents since 13 years old, while the mean age of adolescents is 16.2. This difference implies that their sample has a lower academic level, but more digital technologies use.

All this last information obtained can be analyzed from a complex point of view, following Rhodes et al. (2020), who presented a model to study the pandemic related not only to evidence-based in a social and environmental context, such as policy decisions and interventions. These authors talk about an adaptive science as the new normal, considering that the world is constantly changing, and has to be prepared to emergent contingencies^37^. In this way, several factors can be considered that might condition the results of our study through a more open triangulation approach with multiple data to help make decisions in uncertain situations. More research is needed considering this other way of analyzing our data.

This study evaluated the impact of COVID-19 during the Spanish lockdown concerning insomnia, loneliness, and PA. Descriptive analyses for frequencies and percentages for all variables corresponding to 17 questions from a cross-sectional international survey was completed (Supplemental Material). Bivariate associations examining insomnia, loneliness, and PA were completed. Finally, multiple logistic regression models considering insomnia, loneliness, and PA as dependent variables and as independent variables, age ranges, sex, activity level comparison, or academic education level were completed. The following conclusions were developed from these analyses:

- Females presented a higher insomnia risk, which was greater when decreasing PA levels during lockdown.
- No significant association between PA, insomnia, or loneliness was found.
- A strong correlation between PA and DTS use was observed. Participants with medium and high PA levels utilize more DTS than participants with low PA levels.

The primary contribution of this study is the analysis of insomnia, loneliness, and PA in women during the COVID-19 Spanish lockdown. In the future, the impact of different digital technologic resources on PA participation is needed. It is also important to analyze how age can condition the PA level in case of lockdown. Another interest area needing examination is a comparison of the Spanish data with data obtained from other countries in the global survey.

## Limitations

This study was restricted by the following limitations. First, our main limitation is that the time of MVPA was not included in the study even though it was asked through the IPAQ, because the questionnaire was not well interpreted by participants, as they filled in the questionnaire mixing hours and minutes. No questions were asked concerning pre-pandemic PA levels, and thus, we were not able to compare our data with IPAQ data. Second, our sample may not be generalizable, because a high percent of participants had achieved a high academic level (30% bachelor degree and 44% post-graduate degree). Third, we also did not ask about the type of housing participants lived in, such as whether they lived in a house with a garden or not. All of these could have influenced participant’s PA behaviors. Fourth, sleep disorders for this study were only defined as insomnia and measured using one item. Other unknown sleep problems could have existed.

## Conclusions

We conclude that individuals with low PA levels during lockdown were at great insomnia risk, especially for women. Insomnia risk was lower when not living alone. Active participants utilized more DTS than participants with low PA levels, and women did as well. No significant associations between DTS and age or academic education level were found.

According to the results of this study, we recommend promoting the PA through digital technologies for women. This recommendation would also improve sleep disorders in women who present higher insomnia risks than men.

## Supplementary Information

Supplementary Table 1.

## Data Availability

Anonymized data used and/or analyzed during the current study, along with detailed study protocol, are available from the corresponding authors on reasonable request. The data that support the findings of this study are available from Nationwide Children’s Hospital. but restrictions apply to the availability of these data, which were used under license for the current study, and so are not publicly available. Data are however available from the authors upon reasonable request and with permission of Nationwide Children’s Hospital.
