# Supplementary material for: Impact of COVID-19 lockdown on physical activity, insomnia, and loneliness among Spanish women and men
Source: Sci Rep. 2023 Feb 20;13:2912. doi: 10.1038/s41598-023-30173-2 (PMC9941117; doi:10.1038/s41598-023-30173-2)
Supplement: Supplementary file 1 — Supplementary Table 1. [file 41598_2023_30173_MOESM1_ESM.docx]

**Supplementary material**

**Suppl. Table 1**. Epidemiological features of survey participants

| **Sup.Table 1. Epidemiological features of survey participants** | |  |  |
| --- | --- | --- | --- |
|  | **Males (row%)** | **Females (row%)** | **Total N=996 (total%)** |
| **Sex** |  |  |  |
| **Male** | 288 (100%) | 0 (0%) | 288 (28.9%) |
| **Female** | 0 (0%) | 663 (100%) | 663 (66.5%) |
| **Other** | 0 (0%) | 0 (0%) | 1 (0.10%) |
| **Prefer not to answer** | 0 (0%) | 0 (0%) | 2 (0.2%) |
| **N/A** |  |  | 42 (4.21%) |
| **Age (years):** |  |  |  |
| **18 – 24** | 58 (34,94%) | 106 (63,86%) | 166 (16.7%) |
| **25 – 34** | 52 (29,71%) | 119 (68%) | 175 (17.6%) |
| **35 – 44** | 64 (28,32%) | 154 (68,14%) | 226 (22.7%) |
| **45 – 54** | 55 (25,35%) | 149 (68,66%) | 217 (21.8%) |
| **55 – 65** | 48 (30%) | 103 (64,38%) | 160 (16.1%) |
| **Older 65 years** | 11 (27,5%) | 29 (72,5%) | 40 (4.02%) |
| **N/A** |  |  | 12 (1.20%) |
| **Academic level:** |  |  |  |
| **Elementary School** | 6 (35,29%) | 10 (58,82%) | 17 (1.71%) |
| **Tertiary degree (non-university professional degree)** | 22 (29,73%) | 48 (64,86%) | 74 (7.43%) |
| **High school degree** | 57 (36,31%) | 99 (63,06%) | 157 (15.76%) |
| **Bachelor’s degree** | 70 (23,73%) | 207 (70,17%) | 295 (29.62%) |
| **Post-graduate degree** | 133 (30,57%) | 292 (67,13%) | 435 (43.67%) |
| **N/A** |  |  | 18 (1.8%) |
| **Living by yourself or with others:** |  |  |  |
| **Lived by myself alone** | 34 (43,59%) | 42 (53,85%) | 78 (7.83%) |
| **Live with family or other member(s)** | 248 (27,9%) | 609 (68,5%) | 889 (89.25%) |
| **N/A** |  |  | 29 (2.9%) |
| **Pets:** |  |  |  |
| **Yes** | 14 (31,82%) | 29 (65,91%) | 44 (4.4%) |
| **No** | 40 (30,77%) | 85 (65,38%) | 130 (13.1%) |
| **N/A** |  |  | 822 (82.53) |
| **Covid-19 diagnosed:** |  |  |  |
| **Yes** | 19 (19,59%) | 74 (76,29%) | 97 (9.74%) |
| **No** | 260 (31,14%) | 546 (65,39%) | 835 (83.8%) |
| **Don't know** | 4 (23,53%) | 13 (76,47%) | 17 (1.71%) |
| **Prefer not to answer** | 0 (0%) | 1 (100%) | 1 (0.10%) |
| **N/A** |  |  | 46 (4.62%) |
| **COVID-19 kept me awake at night** |  |  |  |
| **Yes** | 19 (11,38%) | 74 (44,31%) | 167 (16.8%) |
| **No** | 260 (32,1%) | 546 (67,41%) | 810 (81.3%) |
| **N/A** |  |  | 19 (1.91%) |
| **Feeling alone:** |  |  |  |
| **Yes** | 67 (28,51%) | 157 (66,81%) | 235 (23.6%) |
| **No** | 214 (29,6%) | 483 (66,8%) | 723 (72.6%) |
| **N/A** |  |  | 38 (3.82%) |
| **PA level:** |  |  |  |
| **Low activity** | 65 (26,21%) | 168 (67,74%) | 248 (24.9%) |
| **Medium activity** | 55 (30,9%) | 116 (65,17%) | 178 (17.9%) |
| **High activity** | 168 (29,47%) | 379 (66,49%) | 570 (57.2%) |
| **Days walking:** |  |  |  |
| **No walking** | 99 (27,81%) | 240 (67,42%) | 356 (35.74%) |
| **1 day** | 21 (27,63%) | 51 (67,11%) | 76 (7.63%) |
| **2 days** | 29 (30,21%) | 66 (68,75%) | 96 (9.64%) |
| **3 days** | 21 (26,58%) | 54 (68,35%) | 79 (7.93%) |
| **4 days** | 25 (28,74%) | 57 (65,52%) | 87 (8.73%) |
| **5 days** | 12 (19,67%) | 47 (77,05%) | 61 (6.12%) |
| **6 days** | 15 (28,3%) | 37 (69,81%) | 53 (5.32%) |
| **7 days** | 64 (35,96%) | 107 (60,11%) | 178 (17.9%) |
| **N/A** |  |  | 10 (1) |
| **Days of moderate activity:** |  |  |  |
| **No moderate PA** | 126 (27,69%) | 311 (68,35%) | 455 (45.68%) |
| **1 day** | 34 (32,38%) | 69 (65,71%) | 105 (10.54%) |
| **2 days** | 30 (26,09%) | 84 (73,04%) | 115 (11.55%) |
| **3 days** | 31 (26,5%) | 77 (65,81%) | 117 (11.75%) |
| **4 days** | 15 (37,5%) | 21 (52,5%) | 40 (4.01%) |
| **5 days** | 10 (21,28%) | 37 (78,72%) | 47 (4.71%) |
| **6 days** | 15 (39,47%) | 23 (60,53%) | 38 (3.81%) |
| **7 days** | 23 (35,38%) | 35 (53,85%) | 65 (6.53%) |
| **N/A** |  |  | 14 (1.41) |
| **Days doing vigorous PA:** |  |  |  |
| **No vigorous PA** | 132 (26,35%) | 347 (69,26%) | 501 (50.3%) |
| **1 day** | 27 (28,42%) | 66 (69,47%) | 95 (9.54%) |
| **2 days** | 27 (27%) | 69 (69%) | 100 (10.04%) |
| **3 days** | 33 (34,02%) | 58 (59,79%) | 97 (9.74%) |
| **4 days** | 20 (32,79%) | 37 (60,66%) | 61 (6.22%) |
| **5 days** | 23 (38,98%) | 34 (57,63%) | 59 (5.92%) |
| **6 days** | 12 (30%) | 28 (70%) | 40 (4.02%) |
| **7 days** | 12 (34,29%) | 21 (60%) | 35 (3.51%) |
| **N/A** |  |  | 8 (0.8%) |
| **PA level comparison:** |  |  |  |
| **Increased** | 56 (25,93%) | 147 (68,06%) | 216 (21.7%) |
| **Decreased** | 157 (29,24%) | 366 (68,16%) | 537 (53.9%) |
| **Almost the same** | 59 (33,71%) | 109 (62,29%) | 175 (17.6%) |
| **Don't know .../ Hard to tell** | 3 (14,29%) | 17 (80,95%) | 21 (2.11%) |
| **N/A** |  |  | 47 (4.72%) |
| **PA facing difficulties:** |  |  |  |
| **Yes** | 189 (27,96%) | 462 (68,34%) | 676 (67.9%) |
| **No** | 81 (36%) | 137 (60,89%) | 225 (22.6%) |
| **N/A** |  |  | 95 (9.54%) |
| **With whom PA:** |  |  |  |
| **By myself alone** | 169 (33,33%) | 319 (62,92%) | 507 (50.9%) |
| **With family or other member(s)** | 73 (21,6%) | 251 (74,26%) | 338 (33.9%) |
| **N/A** | 46 (30,46%) | 93 (61,59%) | 151 (15.2%) |
| **Used PA programs online:** |  |  |  |
| **Yes** | 121 (20,37%) | 448 (75,42%) | 594 (59.6%) |
| **No** | 148 (42,9%) | 183 (53,04%) | 345 (34.6%) |
| **N/A** |  |  | 57 (5.72%) |
| ***N/A: Not Available; PA: Physical Activity** |  |  |  |
